# Reviewing and identifying amino acids of human, murine, canine and equine TLR4 / MD-2 receptor complexes conferring endotoxic innate immunity activation by LPS/lipid A, or antagonistic effects by Eritoran, in contrast to species-dependent modulation by lipid IVa

**DOI:** 10.5936/csbj.201302012

**Published:** 2013-04-05

**Authors:** Thomas Scior, Christian Alexander, Ulrich Zaehringer

**Affiliations:** a Departamento de Farmacia, Benemerita Universidad Autonoma de Puebla, C.P. 72570 Puebla, Pue., Mexico; b Division of Immunochemistry, Research Center Borstel, Leibniz-Center for Medicine and Biosciences, Borstel, Germany

**Keywords:** Toll-like receptors, MD-2, lipopolysaccharide

## Abstract

There is literature evidence gathered throughout the last two decades reflecting unexpected species differences concerning the immune response to lipid IVa which provides the opportunity to gain more detailed insight by the molecular modeling approach described in this study. Lipid IVa is a tetra-acylated precursor of lipid A in the biosynthesis of lipopolysaccharide (LPS) in Gram-negative bacteria. Lipid A of the prototypic *E. coli*-type is a hexa-acylated structure that acts as an agonist in all tested mammalian species by innate immunorecognition *via* the Toll-like receptor 4 (TLR4)/myeloid differentiation factor 2 (MD-2) receptor complex. In contrast, lipid IVa is proinflammatory in mouse cells (agonism) but it remains inactive to human macrophages and even antagonizes the action of potent agonists like *E. coli*-type lipid A. This particular ambivalent activity profile of lipid IVa has been confirmed in other mammalian species: in equine cells Lipid IVa also acts in a weak agonistic manner, whereas being inactive and antagonizing the lipid A-induced activation of canine TLR4/MD-2. Intriguingly, the respective TLR4 amino acid sequences of the latter species are more identical to the human (67%, 68%) than to the murine (62%, 58%) ortholog. In order to address the unpaired activity-sequence dualism for human, murine, canine and equine species regarding the activity of lipid IVa as compared to LPS and lipid A and, we review the literature and computationally pinpoint the differential biological effects of lipid IVa *versus* LPS and lipid A to specific amino acid residues. In contrast to lipid IVa the structurally related synthetic compound Eritoran (E5564) acts consistently in an antagonistic manner in these mammalian species and serves as a reference ligand for molecular modeling in this study. The combined evaluation of data sets provided by prior studies and *in silico* homology mapping of differential residues of TLR4/MD-2 complexes lends detailed insight into the driving forces of the characteristic binding modes of the lipid A domain in LPS and the precursor structure lipid IVa to the receptor complex in individual mammalian species.

## Structure-activity analysis of lipopolysaccharide immunorecognition by mammalian TLR4/MD-2 complexes

**Figure 1:**
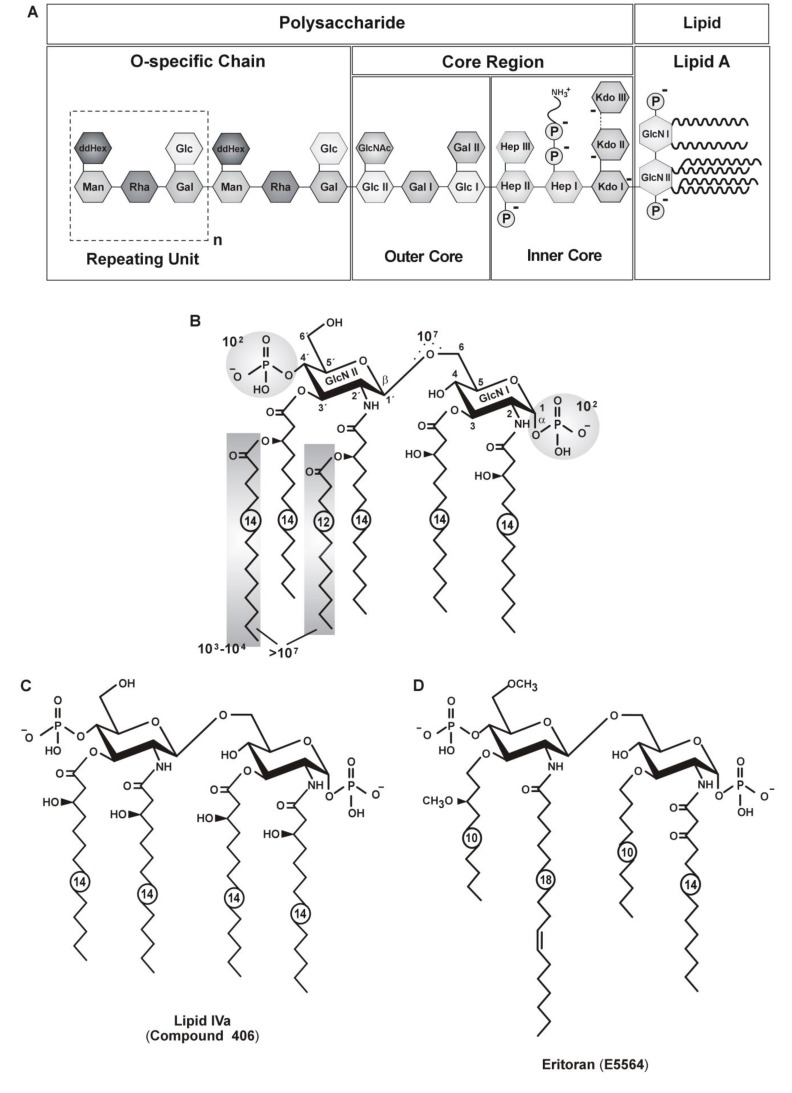
Structures of a typical enterobacterial lipopolysaccharide (LPS), lipid A, precursor lipid IVa (compound 406) and Eritoran (E5564). In their general architecture LPS molecules consist of the membrane-anchoring lipid A domain and an oligo- or polysaccharide region of variable length and chemical composition (panel A). The prototypic E.coli Lipid A shows a hydrophobic region composed of six (hydroxy-) acyl chains of 12 and 14 carbon atoms (panel B). In addition, five experimental values of reduction in human monocyte activation due to the lack of the indicated structural elements are given (panel B). The tetra-acylated biosynthetic precursor Lipid IVa of mammalian LPS/lipid A and its synthetic analogue compound 406 are displayed (panel C) next to the tetra-acyl compound Eritoran (E5564) (panel D). See text for details.

The lipopolysaccharide (LPS) of major commensal Gram negative bacteria such as enterobacteria or *Neisseria* is a powerful activator of innate immunity in mammalian species acting on a picomolar level as pathogen/microbe associated molecular pattern (PAMP/MAMP) *via* molecular recognition of the hexa-acylated and di-phoshorylated lipid A domain by the Toll-like receptor 4 (TLR4) / myeloid differentiation factor 2 (MD-2) receptor complex [1, 2]. The scaffold of prototypic *Escherichia coli-type* lipid A embraces a di-glucosamine di-phosphate backbone with a defined membrane anchor structure of six aliphatic acyl chains (Figure 1). Beside a core-oligosaccharide, LPS typically possesses a variable sugar chain called O-antigen or O-chain (Figure 1). Removing both the core-oligosaccharide and the O-chain yields lipid A still capable of inducing endotoxicity (15 in [3]), even if exceptions have been reported [4].

The innate immune system mediates very effective recognition of invading bacteria on a molecular level by receptor/sensor proteins localized at the cell surface and intracellular sites. Due to this high affinity binding of response triggering bacterial molecules at picomolar concentrations, practical laboratory work is driven to the cutting edge of what can be achieved technically concerning isolation, analysis, purification or contaminants detection. Hence, when interpreting bioactivities of LPS, lipid A and LPS/lipid A substructures it matters if they are obtained from natural sources or chemical synthesis [5]. As revealed in the last two decades, two accessory extracellular proteins, LPS-binding protein (LBP) and CD14 significantly contribute to the extreme sensitivity of mammlian innate immunity to LPS by specific extraction of a single LPS moiety from endotoxin aggregates or bacterial membranes and its transfer to the TLR4/MD-2 heterodimer [6, 7].

In LPS of wild-type enterobacteria an inner and outer core region and the strain-specific O-specific chain have been defined in the polysaccharide region based on evolutionary variation. In a set of specific enterobacterial glycosyltransferase mutants diplaying a rough (R)-type colony form only partial poly/oligo saccharide structures are expressed.

For instance, only the inner and outer core parts are present in Ra-chemotype LPS [8]. The prototypic and also dominant form of *E.coli* Lipid A as represented by its synthetic analog compound 506 consists of a β(1′-6) linked backbone of two monophosphorylated glucosamines designated as GlcN I and GlcN II and a characteristic hydrophobic region composed of six (hydroxyl-) acyl chains of 12 and 14 carbon atoms (Figure 1, panel B). More in detail, within this hexa-acylated structure a subset of four 3-hydroxymyristoyl (3-OH-C_14_) residues is attached directly to the β-D-glucosaminyl-(1,6)-β-D-glucosamine backbone by two amide and two ester bonds at positions 2*/*2′and 3*/*3′, respectively, and the 3-OH-groups of both of the ′primary′ residues at positions 2′and 3′on GlcN II are further esterified to a lauroyl (C_12_) and a myristoyl (C_14_) group, respectively. The two phosphate residues of the lipid A's backbone differ chemically: one is attached in an α-glycosidic linkage to the reducing monomer (GlcN I) in position C1 of the disaccharide scaffold and the other is ester-bound to position 4’ of the nonreducing pyranose unit (GlcN II) [9]. The (-) sign marks the 2 and 6 negative charges in lipid A and the inner core, respectively. Additionally given (Figure 1, panel B) are the approximate numerical values describing the relative reduction of human monocyte activation due to the lack of the indicated structural elements as measured by comparing the *in vitro* cytokine induction activities of the corresponding synthetic partial structures to complete lipid A (compound 506).

As compared to the ubiquitous activation of mammalian TLR4/MD-2 signaling by enterobacterial LPS or lipid A, particular lipid A substructures like the tetra-acylated biosynthetic precursor Lipid IVa or its synthetic analogue compound 406 act either as antagonists or weak receptor agonists in a species-dependent manner (Figure 1, panel C). In contrast, the tetra-acyl compound Eritoran (E5564) acts as a TLR4/MD-2 receptor antagonist in all mammalian species investigated to date (Figure 1, panel D).

**Figure 2:**
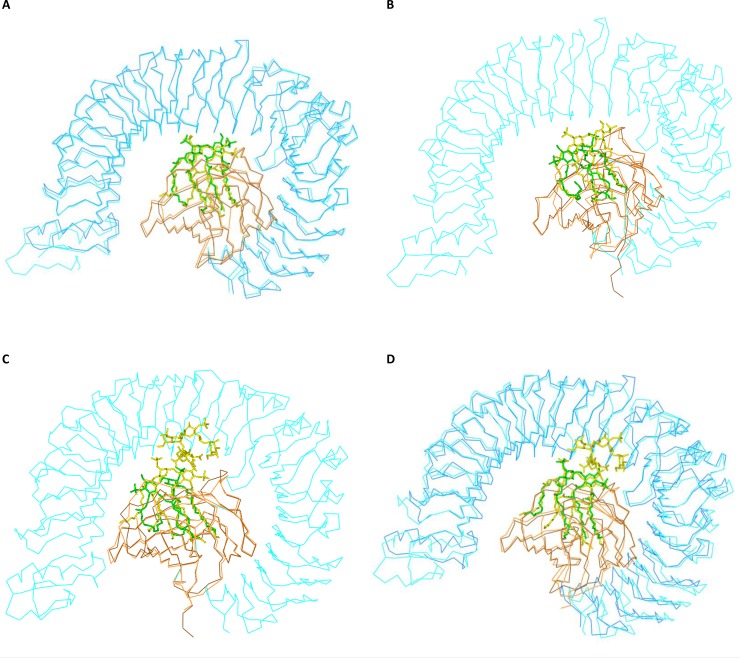
Pairwise superimposition of monomeric units of the murine and human TLR4/MD-2 or MD-2 crystal structures in complex with lipid IVa or LPS/lipid A. (**A**) m(TLR4/MD-2/L4a)2 of 3VQ1 [15] and m(TLR4/MD-2/Re-LPS)2 of 3VQ2 [15] with only lipid A shown. (**B**) m(TLR4/MD-2/L4a)2 of 3VQ1 [15] and h(MD-2/L4a) of 2E59 [11]. (**C**) h(TLR4/MD-2/Ra-LPS)2of 3FXi [8] and h(MD-2/L4a) of 2E59 [11]. (**D**) h(TLR4/MD-2/Ra-LPS)2 of 3FXi [8] and m(TLR4/MD-2 /Re-LPS)2 of 3VQ2 [15] with only lipid A shown. Color codes: Protein backbones are colored in bluish for the solenoid TLR4 ectodomain and in orange/brownish for MD-2 in all panels (A-D). In panel A: 3VQ2 appears darker than 3VQ1; in panel B: 2E59; in panel C: 2E59; and in panel D: 3VQ2 again. Ligands: Lipid IVa: always green in panels A to C; but yellow for the second lipid IVa in m(TLR4/MD-2/L4a)2 of 3VQ1 [15] in panel B; lipid A partial structure of Re-LPS: yellow in panel A and green in panel D. Ra-LPS: yellow (panels C,D).

The lipid A-specific interaction between the complex of the solenoid TLR4 ectodomain and MD-2 with LPS induces a rearrangement and dimerization to an “m-shaped” signaling complex of two TLR4/MD-2/LPS units [8] (Figure 2). This LPS/lipid A-induced formation of the dimeric (TLR4/MD-2/LPS)_2_ complex on the cellular surface constitutes a key step to activate the innate immune system in mammalian species [9, 10]. In addition, X-ray crystal structures of human MD-2 either alone or in an 1:1 association with a partial structure of the human TLR4 ectodomain have shown that the tetra-acylated ligands lipid IVa and Eritoran also bind to the central binding cleft of human MD-2, but in a largely different and thus non-agonistic orientation as compared to the lipid A domain of LPS [11, 12]. Furthermore, x-ray structural data of the radioprotective 105 kDa (RP105) ectodomain/ myeloid differentiation factor 1 (MD-1) complex representing a major negative feed-back-regulatory element of LPS-induced TLR4/MD-2-signaling have revealed a ligand-independent dimer formation of two RP105/MD-1 units in an inverse orientation as compared to the (TLR4/MD-2-LPS)_2_ complex [13, 14].

In general, loss of one or both phosphate groups, underacylation and/or the replacement of single or multiple acyl residues within the characteristic pattern of five n-(hydroxy)myristoyl chains *plus* one n-lauroyl residue by shorter acyl chains lead to partial or total loss of immunoactivity [5, 16–30]. The binding to TLR4/MD-2 is completely lost when both of the two phosphate groups are removed indicating their importance for providing binding affinity in all partial structures of enterobacterial lipid A tested [16, 24, 31]. The activity is, however, preserved when a carboxyl group replaces the phosphate group on LPS [32]. This indicates a major contribution of negative charges in lipid A-binding to TLR4/MD-2 mediated by positively charged amino acids on TLR4 and MD-2. Another example of a lipid A derivative/partial structure with reduced overall negative charge is 4′-monophosphoryl lipid A (MPLA), which has been shown to display markedly reduced TLR4/MD-2 agonistic activity as compared to the diphosphorylated lipid A parent structure. Binding of these ligands to the receptor binding site leads to only partial activation of the TLR4/MD-2-connected intracellular signaling network of as compared to the full agonist (lipid A), thus resulting in incomplete signaling [28]. In comparison to lipid A, 4′-monophosphoryl lipid A (MPLA) lacks one phosphate group and is therefore unable to contact some positively charged residues on the surface of both MD-2 and TLR4 [2, 8]. Recently, it was observed that the presence or absence of the oligosaccharide core segment from *Capnocytophaga canimorsus* LPS modulates endotoxic potency (Table 1). The structural implications were also discussed based on molecular dynamics simulations of the liganded human MD-2 monomer with lipid A from *C. canimorsus* and *E. coli* [4].

**Table 1:** Blockwise listing of structure-activity relationships concerning TLR4/MD-2 complexes and its ligand recognition mechanism.

Key aspects	Bibliographic sources
Review on the advances about pattern recognition receptors (PRR) and other concepts of molecular host response to lipopolysaccharide infections.	[2]
Lipopolysaccharides are very pontent immunoactivating agents from the outer membrane of Gram-negative bacteria. Their lipid A domains represent the substructures responsible for the strong response of vertebrate immune system.	[33, 34]
The major lipid A structure present in *Escherichia coli* LPS mediates maximal immunoactivation/endotoxicity (agonism) in mammalian species. *E. coli*-type lipid A consists of a central diphosphorylated b-1-4-linked-di-glucosamine backbone carrying a characteristic hexa-acylated hydrophobic unit of five n-(hydroxy)myristoyl (C_14_) residues and one n-lauroyl (C_12_) chain. Both phosphates contribute to the dimerization of the TLR4/MD-2/LPS complex. LPS of *Capnocytophaga canimorsus* experiences a 100-fold lower endotoxicity as *E. coli*. The lipid A domain present in *C. canimorsus* LPS has a penta-acylated monophosphorylated di-glucosamine based structure, i.e. it lacks the corresponding 4′ phosphate group of *E. coli*-type lipid A. More in detail, a 2,3-diaminoglucosysl residue (GlcN-3N) is present in *C. canimorsus* lipid A and the remaining phosphate in position 1 of the unaltered glucosamine (GlcN-1) is transformed into a phosphoethanolamine group. It has a core-oligosaccharide attached to its lipid A moiety which possesses two more zwitter-ionic phosphoethanolamine groups and a negatively charged carboxylate group on Kdo. *C. canimorsus* lipid A is 20,000-fold less endotoxic than its lipid A-core oligosaccharide structure. Probably, the missing 4′ phosphate is functionally replaced by aforementioned carboxylate as a prerequisite for lipid A binding to form the MD-2 complex. This is in line with observations (PDB code: 3FXi) that both backbone phosphate groups of *E. coli* LPS contact TLR4 and MD-2 through ion bridges or polar hydrogen bond networking.	[4, 8, 32]
LPS is bound into a symmetrical “m”-shaped homodimer of two TLR4/MD-2 complexes, while antagonistic Eritoran or lipid IVa crystallize in a TLR4/MD-2/Lig or MD-2 complex, respectively. The TLR4/MD-2/LPS multi-protein complex illustrates the remarkable versatility of the ligand recognition mechanisms employed by the TLR family, which is essential for defense against diverse microbial infection.	[11, 12]
Review of pathogen recognition by TLR proteins and the downstream immune response of TLR signaling.	[35]
Despite the stiff appearance made by the horseshoe-like TLR4 ectomain fragments complexed with MD-2 in the LPS- or lipid IVa liganded crystal structures (PDB codes: 3FXI, 3VQ2) show a C-terminal domain rotation upon dimerization relative to the unliganded 1:1 mTLR4/MD-2 structure (PDB code: 2Z64).	[8, 12, 15]
The Phe126 loop of MD-2 undergoes localized structural changes compared to other known human and mouse MD-2. A more comprehensive description of the horseshoe-like TLR4 and beta-cup folded MD-2 structures is found in the original work of two crystallographer groups.	[11, 12]
Single amino acids have been identified to be essential for the agonistic activity of lipid IVa in the murine system by directed mutagenesis on MD-2 by Muroi and Tanamoto in 2006 and on TLR4 by Meng *et al*. in 2010 and Resman *et al*. in 2009. The latter group complemented their work with a computational docking study which in turn is extended in a more systematic manner in the present study.	[7, 36, 37]
The crystal structure of chicken MD-1 (cMD-1) complexed with lipid IVa is compared with MD-2. The ligand flipping of lipid IVa with respect to LPS is also discussed. The bound lipid IVa in cMD-1 displays the same backbone orientation as elsewhere observed LPS bound to hMD-2. In contrast, when complexed with hMD-2, lipid IVa is in a reversed orientation of antagonists 4.	[38]
The antagonists flipped horizontally, i.e. their backbone is rotated with respect to the crystal structure of LPS They are more deeply bound, acyl and amide groups are hidden in MD-2 pocket, no phosphate group interacts with counter-TLR4, no dimerization (two copies of TLR4/MD-2/Lig) takes place.	[8]
Enzymatic removal of the two phosphate groups on the LPS backbone detoxifies LPS through intestinal phosphatases, *i.e*. the host can reduce potentially toxic effects of gram-negative bacteria of the normal bacterial flora of the small intestine.	[39]
Different binding modes for LPS, lipid IVa and Eritoran in the human system. Compared to both tetra-acylated antagonists bound to MD-2, the binding position of agonistic LPS is more solvent-exposed, which in turn enables bridging both TLR4/MD-2/LPS units to form a dimer (TLR4/MD-2/LPS)_2_. The terminal part of the amide-linked fatty acid FA-1 in position 2 on glucosamine GlcN-1 is exposed to the interface between MD-2 and counter TLR4. In LPS the phosphorylated diglucosamine backbone is displaced by 5 Å towards the solvent area. The respective backbones on lipid IVa and Eritoran become less solvent exposed than the lipid A domain of LPS does and are bound in an inverse orientation (horizontal rotation by 180° as compared to lipid A) to the MD-2 pocket in addition. The relative upward displacement of lipid A as compared to both of the antagonists in the human system allows its phosphate groups to assist dimerization by forming ionic interactions with a cluster of positively charged residues in TLR4 and MD-2. The 180° turn formed by the unique cis-double bond in the octadecanoyl chain present in Eritoran provides an analogous binding mode of this particular long chain acyl residue in the MD-2 pocket as compared to the C_14_/C_12_ acyl chain units of lipid A and lipid IVa.	[7, 8, 11, 12]
The binding of a bacterial diphosphoryl moiety like LPS or lipid A into the ectodomain complex can be seen as a functional analogy to intracellular signal transduction by protein phosphorylation evidenced by the lower activity of monophosphoryl lipid A or its loss by unphosphorylated derivatives thereof.	[40]
The phosphorylation status also affects biological activity. The phosphate groups at 1-, and 4’-positions are crucial for the lipid A activity as agonist, *e.g*. monophosphorylated lipid A greatly shows weaker proinflammatory activity. MD-2-mediated ionic interactions between TLR4 and bound lipid A are essential for response activation. For instance, monophosphoryl lipid A of *Salmonella enterica subsp. enterica serovar. Minnesota*, is a variant form of *E. coli* lipid A, and shows only weakly activity at the LPS receptor complex compared to fully phosphorylated lipid A.	[2, 37]
The lipid A domain of LPS interacts with a large hydrophobic pocket of MD-2. Its glucosamine backbone is phosphorylated on either side. The docked pose allows both phosphates to form ion bridges to positively charged side chains on TLR4 or MD-2, all of which contribute to complex formation (see also last table entry).	[8]
The number and length of the acyl chains determine the agonistic property of lipid A. In particular, hexa-acylated lipid A from *E. coli* is a potent agonist for all mammalian cells. In contrast, tetra-acylated lipid IVa, the precursor of E. coli-type lipid A, acts as an agonist only for certain mammalian species.	[41–43]
Endotoxicity (agonism) drops when either phosphate group of the diphosphoryl backbone is deleted.	[31, 44]
LPS with 6 fatty acids (FA) is more deeply buried into the MD-2 pocket than ligands with 5 FA or just 4 FA. Their molecular volume decreases in the same proportions. LPS with two phosphate groups produces higher endotoxic activity than monophosphoryl derivative of lipid A or other LPS forms with just one phosphate group.	[2, 8, 45, 40]
Species-dependency differences: Agonistic activities show hexa-acylated lipid A in human, mouse and horse (*E. coli*), tetra-acylated lipid IVa in mouse and equine (*E. coli*), as well as RsDPLA (*Rhodopseudomanas sphaeroides*, or formerly *Rhodobacter sphaeroides*) in equine and hamster. In contrast antagonists are lipid IVa in human or Eritoran in all three species (hme).	[46, 45, 40]
Lipid A is the substructure of LPS which is responsible for endotoxic effects. The most potent member of the bacterial family of lipopolysaccharides stems from *Escherichia coli* strains. Lipid A with either higher or lower fatty acyl content reduces activity.	[40]
A hexa-acylated form of *Pseudomonas aeruginosa* (Pa) LPS-mediated induces much stronger pro-inflammatory responses in human cells than the corresponding penta-acylated form of Pa-LPS. In contrast, the difference in bioactivity of penta- *versus* hexa -acylated LPS was significantly less expressed in HEK-293 cells transfected with murine TLR4. A chimeric variant of human TLR4 containing a 82 amino acid segment of murine TLR4 (mTLR4: 287 to 368) results in ′murine-type′ reaction pattern in the comparison of penta- and hexa-acylated LPS structures. Note: our inspection of the area on crystal structure 3FXI shows that several of these amino acid residues are positioned in the TLR4 interface contacting the hydrophilic ligand backbone above the MD-2 pocket and also parts of the dimerization interface of counter-TLR4.	[47]
Both phosphates and hexa-acylation of the lipid A backbone are absolutely required for endotoxicity while hypo-acylatation weakens cell activation. For instance, lipid A present in *Pseudomonas aeruginosa* LPS is penta-acylated with shorter lengths. Precisely, the deletion of just one fatty acids of lipid A results in a complete endotoxicity loss.	[2, 36, 37]
Certain derivatives of LPS were identified as inhibiting agents or agonists according to the applied cell tests. The existence of traces of the highly potent LPS could lead to false positive activity observations in analogy to the residual presence of lipoproteins in reported cases of TLR2-activating LPS preparations. Meanwhile, the situation has improved with the advent of newer analytical technologies to identify and purify smallest amounts of test substances in the picomolar range. A further complication in understanding lipid A biology is its heterogeneity due to natural variations like penta or hexa-acylated forms found in lab strains. Many experiments use lipid A from lab strains that contain a structural mixture (number and length of lipid chains). LPS isolated from *Porphyromonas gingivalis* is highly heterogeneous, which possibly accounts for reports that *P. gingivalis* LPS can activate two innate immune receptors simultaneously. Inflammatory response by Lipid A from *Pseudomonas aeruginosa* is less than that of enterobacterial *E. coli* LPS.	[2, 3, 30]
Review about the mechanisms of TLR-mediated control of innate and adaptive immune system. After aligning onto their common diglucosamine backbones, both antagonists of the human system appear horizontally rotated around 180° (i.e. flipped) with respect to complexed LPS.	[48]
All fatty acids of both antagonists are docked deeply into the hydrophobic binding pocket of MD-2. Quite in contrast, the LPS complex shows an unburied primary fatty acid chain (FA-1 of LPS) not observed in both antagonist complexes.	[7, 8]
The importance of the number of acyl chains for the activity of LA-like structures is illustrated by the properties of a library of synthetic monosaccharide lipid A mimetics (the aminoalkyl glucosaminide phosphate compounds) in which both the number and carbon length of the acyl chains are crucial to generate an active compound. The explanation for why hypo-acylated lipid A causes less cell activation than hexa-acylated lipid A is likely to involve an alteration to the interaction between lipid A and the LPS receptor complex, such that receptor activation is reduced. Both the number and the length of the acyl chains are essential for the full agonist activity of LA. In fact, the production of an under-acylated lipid A and resulting evasion of innate immunity may be associated with virulence in pathogens such as *Yersinia pestis*. *E. coli* hexa-acylated lipid A acts as a pan-agonist for all mammalian cells that express a complete LPS receptor complex. The precursor of *E. coli* lipid A, tetra-acylated lipid IVa, is only an agonist for some species of mammals.	[2, 37, 46, 48, 51, 75]
Review about the TLR2 agonist activities of certain putative bacterial compounds is most likely due to contaminating highly active natural lipoproteins and/or lipopeptides. Endotoxicity strength of LPS with 6 fatty acids (FA) is larger than other derivatives with 5 FA or others with just 4 FA. The acyl chain length is longer on LPS from *E. coli* than that of *Pseudomonas aeruginosa*.	[30]

## Site-directed mutagenesis data of liganded TLR4/MD-2 complexes

Various lipid A derivatives or analogous agents (Figure 1) with a common amphipathic glycolipid scaffold have been described to represent LPS/lipid A-like activators (agonists) or TLR4/MD-2 signaling or inhibitors (antagonists) of LPS-induced cellular immunoactivities [2, 25, 28, 49, 50]. LPS receptor inhibitors are being developed as potential drugs for adjunct treatment of septic shock patients with Gram-negative septic infection (endotoxicity). Lipid IVa, a tetra-acylated lipopolysaccharide precursor in the biosynthesis of *Escherichia coli* or *Salmonella* lipid A, is an agonist in murine myeloid cells but it remains inactive to human macrophages and even antagonizes the action of potent agonists like *E. coli*-type hexa-acylated LA. In contrast to lipid IVa, however, the synthetic compound Eritoran that also comprises of only four acyl chains is a potent TLR4/MD-2 receptor antagonist in human, murine and equine species and can be considered as an investigational drug against bacterial sepsis [2], (48,51,75 in [46]).

Site directed mutagenesis approaches have revealed involvement of species-specific residues in MD-2 [36] and TLR4 in agonistic/antagonistic activities of lipid IVa (Table 2) [8, 37, 51, 52]. Hence these mutagenesis data indicate, that both MD-2 and TLR4 contribute to the species-specific response to lipid IVa [12, 15, 36, 37, 53, 54]. For example Thr57, Val61, and Glu122 of mouse MD-2 may have an impact on activation of the murine receptor complex by lipid IVa [36]. TLR4 was also subject to a set of selective human/horse sequence conversion mutants like R385G [51, 52].

**Table 2:** Listing of experimental findings concerning the amino acid sequences, as well as site directed mutagenesis research describing MD-2 and TLR4 protein involvement.

Article	Used observations	Ref
Diphosphoryl lipid A obtained from the nontoxic lipo-polysaccharide of *Rhodopseudomona ssphaeroides* is an endotoxin antagonist in mice.	RSLA blocks LPS activity of *E. coli in vitro* and *in vivo* (mouse organism). Description of SAR: lipid A of is the active part of LPS. In general, lipid A with higher or lower FA content and a single phosphate group is less active.	[40]
Lipopolysaccharide from *Rhodobacter sphaeroides* is an agonist in equine cells.	In equine monocytes Rs-LPS is a potent agonist of tumor necrosis factor (TNF) production but it inhibits the response to LPS of *E. coli* in a human monocytic cell line.	[45]
Structural regions of MD-2 that determine the agonist-antagonist activity of lipid IV A.	The species-specific activity of lipid IVa reflects species differences in mouse and humanMD-2 structures. Amino acid regions 57-79 and 108-135 determine the species-specific activity of lipid IVa. The replacement of Thr57, Val61, and Glu122 of mMD-2 with corresponding hMD-2 residues Ser57, Leu61 and Lys122 or alanines impaired the mouse-type agonistic activity of lipid IVa, and human-type antagonistic activity became evident.	[36]
Elucidation of the TLR4 / MD-2 interface required for signaling by lipid IVa.	Human residues 57 to 107 changed into the equivalent horse sequence in hMD-2 confer agonist activity of lipid IVa. Conversely, when replacing residues 57-66 and 82-89 on eMD-2 by the corresponding human residues confers agonist activity of hMD-2. Concerning TLR4 a single point mutation changing one horse into a human residue (Arg385Gly) reduced the partial agonist activity of horse toward human response (antagonism).	[52]
Essential roles of hydrophobic residues in both MD-2 and Toll-like receptor 4 in activation by endotoxin.	The role of acyl chains of lipid A and lipid IVa, in particular, FA1 in the interface of human and murine MD-2 counter TLR4, respectively.	[8, 15]
MD-2-mediated ionic interactions between lipid A and TLR4 are essential for receptor activation.	Murine agonist activity is recovered on an mMD-2 / hTLR4 upon single, double or triple mutations at the dimerization interface of the TLR4 in the wedge: hGlu369B into mLys367B, and hGln436B into mArg434B or hLys388B, into mSer386B.	[51]
Essential roles of hydrophobic residues in both MD-2 and toll-like receptor 4 in activation by endotoxin.	In the human TLR4 sequence, Phe440 and Phe463 of counter TLR4 (B chain) as well as three hydrophobic amino acids Val82, Met85, and Leu87 of MD-2 (C chain) together with a fatty acid side chain of hexa-acylated lipid A form a hydrophobic interface between TLR4/MD-2 and counter-TLR4 for innate immune activation. Mutations of Phe440A or Phe463A render TLR4 unresponsive, whereas the Phe440Trp mutant retained full activity. B chain Lys388 is considered irrelevant in contrast to reports by Meng *et al*.	[8, 37]
MD-2 residues tyrosine 42, arginine 69, aspartic acid 122, and leucine 125 provide species specificity for lipid IVa.	MD-2 in presence of TLR4 was found necessary for lipid IVa signaling activity in the mouse system. Site-directed mutagenesis studies show Tyr42, Arg69, Glu122 and Leu125 of MD-2 infer lipid IVa species specificity. Lysine in position 122 of mMD-2 (Glu122C) was mutated into glutamate (Lys122Glu) reducing lipid IVa agonist activity. The dimerization interface was also described.	[51]

## Dimerization and activities in crystal structures

With the 2012 release of the crystal structures of the mouse TLR4/MD-2/L4a and TLR4/MD-2/Re-LPS complexes (PDB codes: 3VQ1, 3VQ2) [15] it became possible to compare two complementary functional pairs (Table 3): (i) agonistic dimeric murine (TLR4/MD-2/L4a)_2_ [15] *versus* the antagonistic monomeric human (MD-2/L4a)1 complex [11]; as well as (ii) agonistic dimeric human (TLR4/MD-2/Ra-LPS)_2_ [8] *versus* the antagonistic monomeric human (MD-2/L4a) complex [11].

**Table 3:** Listing of available crystal structures concerning the biological unit of TLR4/MD-2.

PDB Code Year Ref	Species ( Components in complex) TITLE	Resolution (Å)
**2E59**	Human (MD-2 / L4a)_1_	
2006	CRYSTAL STRUCTURE OF HUMAN MD-2 IN COMPLEX WITH LIPID IVA	2.21
[11]		
**2E56**	Human (MD-2)_1_	
2006	CRYSTAL STRUCTURE OF HUMAN MD-2	2
[11]		
**2Z62**	Human (TLR4 fragment)_1_	
2007	CRYSTAL STRUCTURE OF THE TV3 HYBRID OF HUMAN TLR4 AND HAGFISH VLRB.61	1.7
[12]		
**2Z63**	Human (TLR4 fragment)_1_	
2007	CRYSTAL STRUCTURE OF THE TV8 HYBRID OF HUMAN TLR4 AND HAGFISH VLRB.61	2
[12]		
**2Z64**	Mouse (TLR4 fragment / MD-2)_1_	
2007	CRYSTAL STRUCTURE OF MOUSE TLR4 AND MOUSE MD-2 COMPLEX	2.84
[9]		
**2Z65**	Human (TLR4 / MD-2 / Eri)_2_	
2007	CRYSTAL STRUCTURE OF THE HUMAN TLR4 TV3 HYBRID-MD-2-ERITORAN COMPLEX	2.7
[12]		
**2Z66**	Human (TLR4 fragment)_4_	
2007	CRYSTAL STRUCTURE OF THE VT3 HYBRID OF HUMAN TLR4 AND HAGFISH VLRB.61	1.9
[12]		
**3FXI**	Human (TLR4 / MD-2 / Ra-LPS)_2_	
2009	CRYSTAL STRUCTURE OF THE HUMAN TLR4-HUMAN MD-2-E.COLI LPS RA COMPLEX	3.1
[8]		
**3MU3**	Chicken (MD-1 / L4a)_2_	
2010	CRYSTAL STRUCTURE OF CHICKEN MD-1 COMPLEXED WITH LIPID IVA	2.4
[13]		
**3MTX**	Chicken (MD-1 / - * -)_2_	
2010	CRYSTAL STRUCTURE OF CHICKEN MD-1	2
[13]	(* but MD-1 is liganded)	
**3VQ1**	Mouse (TLR4/MD-2/L4a)_2_	
2012	CRYSTAL STRUCTURE OF MOUSE TLR4/MD-2/LIPID IVA COMPLEX	2.7
[15]		
**3VQ2**	Mouse (TLR4/MD-2/LPS)_2_	
2012	CRYSTAL STRUCTURE OF MOUSE TLR4/MD-2/LPS COMPLEX	2.48
[15]		

The oligomerization state is different for the liganded and unliganded complexes and it also depend on the environment: In 2007 it was described that LPS binding induces the dimerization of hTLR4/MD-2 [55]. Crystallography (Table 3) showed unambiguously the dimeric structures of human (TLR4/MD-2/LPS)_2_ [48] and mouse (TLR4/MD-2/LPS)_2_ complexes [15]. In native gel electrophoresis experiments the complex was shown to be a dimer: m(TLR4/MD-2/Re-LPS)_2_. In contrast, when bound to lipid IVa the complex remained monomeric m(TLR4/MD-2/L4a) in solution according to the electrophoretic mobility shift data. However, the lipid IVa murine complex was dimeric in the crystal form: (TLR4/MD-2/L4a)_2_ [15]. The authors ascribe its missing dimerization in solution to weaker noncovalent forces in the dimerization interface, assuming that the dimerization would be enhanced in the membrane where movements are restricted to the cell surface to facilitate contacts. If this holds, then the structural events as observed by crystallography of mouse (TLR4/MD-2/L4a)_2_ and human MD-2/L4a monomer are in close keeping with the biological function of either agonism (mouse) or antagonism (in human cells).

As pointed out by Park and colleagues [8] the initial comparison of the human dimeric (TLR4/MD-2/Ra-LPS)_2_ crystal structure (PDB code: 3FXI) with the monomeric antagonist complexes of lipid IVa with human MD-2 (PDB code: 2E59) and Eritoran with a TLR4/MD-2 fragment (PDB code: 2Z65), revealed that the presence of the two additional (secondary) acyl residues in the lipid A domain of Ra-LPS is correlated with a relative upward shift of the di-phosphorylated glucosamine backbone by approximately 5 Å towards the solvent area. This structural shift allows phosphate groups of LPS to contribute to receptor multimerization by forming ionic interactions with a cluster of positively charged residues in human TLR4 and MD-2. The results of the pairwise superimposition of this set of four crystal structures (Table 4) were visualized (Figure 2).

**Table 4:** Correlations between ligand orientation, its position and function are listed for available crystal structures. First two columns show the complexes with the ligands. Third column shows their interrelatedness according to position and effects. Last column shows the relative orientation of the glucosamine backbone of LPS, lipid A or lipid IVa. LPS in 3FXI [8] serves as a reference with its backbone orientation in the MD-2 pocket arbitrarily designated as “fit”, and the inverse orientation of the di-phosphorylated di-glucosmine backbone i.e. the binding mode in an 180° horizontal backbone rotation is termed “flipped”. The corresponding pairwise superimpositions of monomeric units of the murine and human TLR4/MD-2 or MD-2 crystal structures in complex with lipid IVa or LPS/lipid A in are shown in Figure 2.

PDB; complex with ligand L1	PDB; complex with ligand L2	L1 *vs* L2: Positions: above / below or equal; Effect 1: Effect 2	L1 *vs* L2: Orientations: fit= backbone positioning as LPS in 3FXI [8]
3VQ1; dimer:	3VQ2; dimer:	L4a__LPS;	fit__fit
m(TLR4/MD-2/L4a)_2_	m(TLR4/MD-2/LPS)_2_	AG: AG	
3VQ1; dimer:	2E59; monomer:	L4a / L4a;	fit / flipped
m(TLR4/MD-2/L4a)_2_	h(MD-2 /L4a)	AG: AN	
3FXi; dimer:	2E59; monomer:	LPS / L4a;	fit / flipped
h(TLR4 /MD-2 /Ra-LPS)_2_	h(MD-2 L4a)	AG: AN	
3FXi; dimer:	3VQ2; dimer:	LPS**__**LPS;	fit**__**fit
h(TLR4 /MD-2 /Ra-LPS)_2_	m(TLR4/MD-2/LPS)_2_	AG: AG	

## Dimerization and signaling

Mechanistically different aspects of signaling are in discussion: ligand-induced oligomerization, cytoplasmically driven self-association or agonistic dimerization [15, 20, 56]. LPS binding to the cell surface receptor TLR4 constitutes the extremely specific and effective agonistic stimulus for transmembrane signaling *via* connecting Toll/IL-1R endodomain (TIR) to mount an immediate immune response. Intriguingly, recent experiments on replacement of the TLR4 ectodomain with MD-2, CD14, monomeric fluorescent protein or a 24 kDa gyrase B fragment revealed a robust ligand-independent constitutive activation of signaling by the corresponding chimeric fusion proteins, comparable to the maximal stimulation of the receptor with LPS. As discussed by the authors this indication for an intrinsic dimerization propensity of the transmembrane and cytoplasmic domains of TLR4 and reveals a previously unknown function of the ectodomain in inhibiting spontaneous receptor dimerization, since the unliganded TRL4 receptor complex must be kept in an inactive state without release of undesired immune responses [57].

In 2009, Meng *et al*. published mutational observations about important residues on the TLR4/MD-2 protein complex which were animated through our SPL scripting in the 3D models (Table 5) [37]. Surprisingly, the equine residues are identical with human residues contrary to what could be expected from their activity differences concerning lipid IVa. It acts as an antagonist in human cell tests but as a full or partial agonist in mouse or horse systems, respectively.

**Table 5:** The first two rows in each of the three blocks list the residues present in the double mutant postions shown to interchange human and mouse activities of lipid IVa on the TLR4/MD-2 protein complex [37]. The third line of each block adds the equivalent amino acids of the horse protein (eTLR4).

Species	absolute residue id in complex	equivalent amino acids in TLR4*	known L4A activities	known LPS activities	known Eritoran activities
H	1011	GLN436	AN	AG	AN
M	1282	ARG434	AG	AG	AN
E	1289	GLN437	partial AG	AG	AN
H	963	LYS388	AN	AG	AN
M	1234	SER386	AG	AG	AN
E	1241	LYS389	partial AG	AG	AN
H	944	GLU369	AN	AG	AN
M	1215	LYS367	AG	AG	AN
E	1222	GLU370	partial AG	AG	AN

More in detail, the backbones substructures of agonists were consistently found to display an orientation with the reducing glucosamine-1-phosphate unit facing the secondary (signaling) dimerization site at the open side of the MD-2 pocket, whereas the antagonists were consistently crystallized in the inverted backbone orientation, rotated (′flipped′) by 180° as compared to the agonistic ligands. Moreover, the hydrophilic backbone structures of all agonistic ligands have been found to be placed in a significantly upward shifted position relative to MD-2 surface as compared to the antagonists.

Now, it is to say, that contrary to classical pharmacology, where drugs act as either (full or partial) agonists or antagonists, lipid IVa acts either as an antagonist or an agonist in a characteristic species-dependent manner. Evidently, there is not a corresponding ′classical′ structure-activity relationship for lipid IVa, since the ligand structure is the same. However, a correlation has been revealed between the species-characteristic agonist *versus* antagonist activities of lipid IVa and the overall binding mode this particular ligand i.e. how deep lipid IVa is buried in the MD-2 pocket and which mode of backbone orientation is present along the binding cleft. Apparently, when this tetra-acylated ligand binds very deeply into the pocket, then the specific unit of one phosphate group (and/or any acyl equivalent nearby, KDO) is not exposed on the dimerization side of the TLR4/MD-2/L4a monomer in order to attract a second monomer. This species-related TLR4/MD-2/L4a monomer thus fails in attracting a second monomer to initiate dimerization and consecutive downstream signaling.

In this view, lipid IVa appears to be rather an imperfect agonist than an agent with dual activity. As a comparably lower affinity ligand, it can bind in species-dependent manner either in an agonistic orientation (with its more surface-exposed backbone as found in the murine receptor complex) or the antagonistic orientation (with the flipped or inversed di-phospho-di-glucosamine backbone as revealed for the monomeric human MD-2 complex).

The earlier reviewed literature attests that lipid IVa is an agonist in mouse but acts as an antagonist in human cells. It can be assumed, that binding of agonists lead to a dimerization of liganded Toll-like receptor 4 and myeloid differentiation factor 2 complexes which then triggers downstream signaling for anti-inflammatory response. Cytoplasmically-driven self-association was, however, also reported.

**Figure 3:**
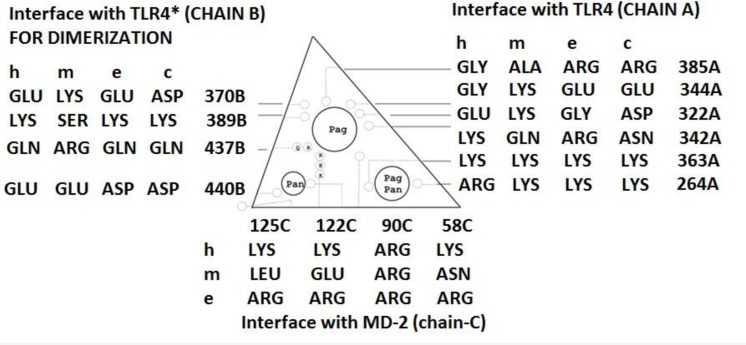
Schematic view of the wedge, a triangular space between the three interacting polypeptide chains, A, B and C corresponding to TLR4, secondary or counter-TLR4 (labeled TLR4*) and MD-2 proteins, respectively. The numbering of the TLR4 residues refers to the horse sequence. Due to deletions of a few TLR4 residues their numbers differ slightly at equivalent positions: up to equine position number 297: e = h=c = m + 1; then from equine position 298 to 560: e = m + 3 = h + 1 = c + 1 or h = m + 2 = e-1 = c-1. For instance, comparison of the residues present in (equine) position 322A indicates repulsion effects for the agonistic phosphate group (Pag) of lipid IVa in human (Glu-321A) and canine (Asp-321A) complexes, but strong attraction for murine (Lys-319A) and less for equine (Gly-322A) systems. In mice, the positions 367B and 434B push the phosphate group into Pag. Position 367B destabilizes Pag as phosphate localization in human, equine and canine systems. On the wedge bottom, MD-2 has a cation-reach vestibule to accommodate (Pag-rejected) phosphate groups in Pan except for mice (mGLU122C). Surrounded by conserved residues, Pag/Pan always accommodates the other (second) phosphate group of lipid IVa. The Pan position is occupied by the GlcN II phosphate group of lipid IVa or Eritoran, while the GlcN I phosphate group of LPS / lipid A occupies Pag. In the highly conserved right corner of the wedge is the all species-shared Pag-Pan site holding the complementary phosphate groups of the backbones: either GlcN I phosphate group for lipid IVa and Eritoran, or GlcN II phosphate group for LPS and lipid A.

Based on the x-ray crystal structures of dimeric TLR4/MD-2*agonist complexes the presentation of the three protein chains in the region of the dimerization interface, i.e. TLR4 (chain A), MD-2 (chain B) and TLR4* (chain C, ′counter′ TLR4) as a triangular interaction zone (“wedge”) is a most useful concept to analyze species differences and to predict mutational effects (Figure 3). In this structural selection the wedge-like area consists of three sides: two partial structures of TLR4 proteins contributing to the ′primary′ and ′seondary′dimerization interfaces and one MD-2 forming the connecting bottom line. On a molecular level the species-dependent activity profiles of lipid IVa are reflected by a concert of conserved and variable amino acids in their respective protein sequences in this wegde-shaped zone.

## Dimerization and activity

The differential height of the phosphates above the wedge bottom (MD-2) directly reflects the activity changes between the species. Only a highly exposed phosphate group of the backbone is capable of linking two liganded TLR4/MD-2 units [8]. Hence, this site (labeled as “Pag” in Figure 3) is considered to play the key role for switching to agonism (Pag) from antagonism (Pan) and back. While the Pag site contacts the GlcN I phosphate group of all observed agonists, the Pan site interacts with the GlcN II phosphate group of all antagonists as the latter show a flipped backbone.

According to the binding models the equine residue pair eGly322A & eGlu344A at the TLR4 interface corresponds to hGlu321A & hGly343A or mLys319A & mLys341A of the human and murine systems, respectively (Figure 3). The former two pairs are interrelated in a homologous way. The murine pair differs by two cationic residues and stabilizes the phosphate group in its agonistic site. Moreover, this pair together with others (nonconserved hGly384 vs. mAla382 vs. eArg385) nicely explain the crystallographically observed shift in the wedge's leftmost phosphate groups from a MD-2 assisted antagonistic phosphate binding site (interacting hARG90C) toward the agonistic phosphate site.

Park *et al*. already mentioned the relevant phosphate bridging of liganded TLR4/MD-2 unit (chains A,C) toward a second or counter-TLR4 (chain B) in the wedge, a concert of interacting residues assists the dimerization interface (Figure 3).It can be assumed that homodimerization is signaling relevant, since agonistic lipid IVa appears as (TLR4/MD-2/L4a)_2_ in mouse complexes [14] which parallels agonistic LPS bound to mouse and human crystal complexes [8, 14]. Very close to the wedge lies a histidine-rich surface patch (never referred to) in the dimerization interface of the two TLR4 proteins (chain A and chain B of 3D template 3FXI [8], which is the counter TLR4, sometimes labeled as TLR4* in the literature). This dimerization interface probably plays a role in transmembrane signal transduction.

## Conclusions

During eons only LPS has been relevant for evolutionary adaptation. During time any random mutations were kept in the genes if they were not detrimental to the LPS recognition (silent point mutations) with the present day consequence that all nonidentical residues in MD-2 and TLR4 sequences do not affect picomolar LPS recognition. However, this is not the case of synthetic lipid IVa which is also a biosynthetic interim in bacterial cells. The hitherto silent mutations for LPS in mammalians become relevant for lipid IVa. Mapping makes the interacting net of amino acids recognizable (Figure 3). The homodimerization process in mice is helped by a nonconserved nonpolar residue (mAla414B) sitting in opposition to what would be the antagonistic phosphate (Pan) on (counter) TLR4*. Thanks to the nonpolar alanine - anionic repulsion, the phosphate group becomes a theoretical “rejection group” and the phosphate group moves into its agonist position further up as an accessible alternative, which actually is the only solution in the wedge. In its linear elongated conformation the mArg434B can interact with the phosphate group in agonist position. This amino acid is not conserved in horse, but compensated through the presence of eArg385A which moves the phosphate group into an “in-between pose” between aforementioned agonistic phosphate in the upper left corner of the wedge and its antagonistic counterpart in its lower left corner.

Taken together the collected data in a more general view, binding of agonists to TLR4/MD-2 leads to formation of a dimeric (TLR4/MD-2/Ligand)_2_ complex that efficiently activates downstream signaling to activate the vertebrate innate immune response while antagonists act as competitive inhibitors of agonists by binding to the monomeric TLR4/MD-2 unit in a non-dimerizing and thus non-signaling, hence, unproductive manner. Drawn from the hitherto known crystal structures, there is a complex correlation between binding and biological function regarding the exact ligand positioning and in particular the stretched diphophosphorylated di-glucosamine backbone spanning the width of the MD-2 unit reaching from one TLR4 to the other (counter) TLR4* in view of the changing agonistic *versus* antagonistic activities of complexed ligands. All agonist ligands have been shown to mediate a specific bridging of two TLR4/MD-2 subunits to dimeric complexes (TLR4/MD-2/Lig)_2_ in the crystal structures whereas the antagonists apparently do not provide the dimerization of TLR4/MD-2 complexes. A crucial finding is the recognition of a murine acidic residue in the otherwise basic vestibule of MD-2. It leads to a repulsion and phosphate shift into agonist position which enables the ligand backbone to bridge both TLR4/MD-2 units. The right corner of the wedge (Figure 3) is highly conserved and holds the GlcN II phosphate group of agonists or, GlcN1 phosphate of antagonists due to their flipped backbones. This is why liganded TLR4/MD-2 forms most likely the functional biological unit. Hence, it must be considered a “monomer” (strangely, a heteromonomer, so to speak). It constitutes the “attachment point” or devise for the association of another liganded TLR4/MD-2 unit to form the signaling dimer.

## Glossary and terminology

*: Asterisk marks counterpart in dimer complex,
/: Interface symbol linking protein subunits TLR4, TLR4*, MD-2 or MD-2*
ABCD: Chain A = TLR4, B = TLR4*, C = MD-2, and D = MD-2*
AG, ag: Agonist
AN, an: Antagonist
dimer: Dimerization of two monomeric units (MD-2/TLR4) to form a homodimer (MD-2 / TLR4)_2_ complex
GlcN: glucosamine of the diglucosamine backbone of LPS
hmec: Four investigated species: c = Dog, e = Horse, m = Mouse, and h = Human
L4a: Lipid IVa (only used in formulae)
monomer: Heterodimeric unit of two chains (A and C) into a functional MD-2/TLR4 complex, sometimes refered to as the heterodimeric subunit in the literature.
P: Phosphate group(s) on three sites in the wedge, cf. Figure 3
PDB: Protein Data Bank entry (http://www.rcsb.org/pdb/)
wedge: triangular empty space between TLR4, MD-2 and TLR4*
